# Endoglin and TGF-β signaling in glioblastoma

**DOI:** 10.1007/s00441-020-03323-5

**Published:** 2021-01-20

**Authors:** Isabel Burghardt, Elisa Ventura, Tobias Weiss, Judith Johanna Schroeder, Katharina Seystahl, Christian Zielasek, Dorothee Gramatzki, Michael Weller

**Affiliations:** Laboratory of Molecular Neuro-Oncology, Department of Neurology, University Hospital and University of Zurich, Zurich, Switzerland

**Keywords:** Endoglin (CD105), Glioblastoma, Signaling, SMAD, TGF-β

## Abstract

Microvascular proliferation is a key feature of glioblastoma and neovascularization has been implicated in tumor progression. Glioblastomas use pro-angiogenic factors such as vascular endothelial growth factor (VEGF) for new blood vessel formation. Yet, anti-VEGF therapy does not prolong overall survival so that alternative angiogenic pathways may need to be explored as drug targets. Both glioma cells and glioma-associated endothelial cells produce TGF-β superfamily ligands which bind TGF-β receptors (TGF-βR). The TGF-βR type III endoglin (CD105), is a marker of proliferating endothelium that has already been studied as a potential therapeutic target. We studied endoglin expression in glioblastoma tissue and in glioma-associated endothelial cells in a cohort of 52 newly diagnosed and 10 recurrent glioblastoma patients by immunohistochemistry and by ex vivo single-cell gene expression profiling of 6 tumors. Endoglin protein levels were similar in tumor stroma and endothelium and correlated within tumors. Similarly, endoglin mRNA determined by ex vivo single-cell gene expression profiling was expressed in both compartments. There was positive correlation between endoglin and proteins of TGF-β superfamily signaling. No prognostic role of endoglin expression in either compartment was identified. Endoglin gene silencing in T98G glioma cells and in human cerebral microvascular endothelial cells (hCMEC) did not affect constitutive or exogenous TGF-β superfamily ligand-dependent signaling, except for a minor facilitation of pSmad1/5 signaling in hCMEC. These observations challenge the notion that endoglin might become a promising therapeutic target in glioblastoma.

## Introduction

Glioblastoma is the most prevalent and malignant primary brain tumor and remains essentially fatal despite current multi-modality therapy (Weller et al. 2017). Glioblastoma is a highly vascularized tumor. There is the notion that a proportion of glioblastoma vasculature is neoplastic as glioblastoma stem cells may differentiate into CD31-positive endothelial cells or $\alpha$smooth muscle actin (SMA)-positive pericytes (Das and Marsden 2013). Yet, treatment with the monoclonal antibody bevacizumab targeting the pivotal proangiogenic factor vascular endothelial growth factor (VEGF) has not improved overall survival of glioblastoma patients in recurrent (Wick et al. 2017) or newly diagnosed glioblastoma (Chinot et al. 2014; Gilbert et al. 2014) or on a population level (Gramatzki et al. 2018). This indicates that multiple factors may participate in glioma neoangiogenesis or that its inhibition may not affect overall outcome.

TGF-β signaling is regulated in a complex, coordinated manner at multiple levels (ten Dijke and Arthur 2007). In cultured glioma cells, transforming growth factor (TGF)-β regulates VEGF secretion via Smad2/3- and Smad1/5/8-dependent signaling (Seystahl et al. 2015). TGF-β signaling has also been reported to depend on extracellular matrix proteins such as latent TGF-β binding protein (LTBP)-1 (Tritschler et al. 2009) and LTBP-4 (Wang et al. 2016). The key mediators of TGF-β signaling, however, include various TGF-β receptors. TGF-β interacts with three major receptor classes termed TGF-βR-I-III. Type I and type II receptors are transmembrane receptor serine/threonine kinases. Upon TGF-ß ligand binding, heteromeric complexes of TGF-βR I and II recruit the Smad proteins. Generally, the pathway has been described to mainly split into two distinct branches downstream of type I receptors, which are also known as activin receptor-like kinases (ALK) (Miyazawa et al. 2002). ALK-4, ALK-5, and ALK-7 specifically phosphorylate Smad2 and Smad3, whereas ALK-1, ALK-2, ALK-3, and ALK-6 specifically phosphorylate Smad1, Smad5, and Smad8 (Schmierer and Hill 2007), with ALK-5 also being involved in the regulation of Smad1- and Smad5-dependent signaling. TGF-βR heterodimeric complexes may also directly interact with or phosphorylate non-Smad proteins initiating parallel signaling that cooperates with the Smad pathway. The molecules which participate in non-Smad TGF-β signaling include mitogen-activated protein kinases (MAPK)/ERK, p38, and c-Jun N-terminal kinases (JNK) (Moustakas and Heldin 2005) and phosphoinositide 3′ kinase (PI3K)/Akt (Zhang et al. 2013).

In addition to the TGF-β type I and type II receptors, the TGF-β receptor core complex may involve TGF-β type III receptors. The type III receptor endoglin (also known as CD105) is expressed as a homodimeric membrane-anchored proteoglycan being stabilized by disulfide bonds. Endoglin shedding through proteolytic cleavage of the extracellular domain (ECD) of the receptor releases a soluble form of endoglin. Soluble endoglin has been observed in serum of pregnant women with preeclampsia (Venkatesha et al. 2006) and in the serum of cancer patients (Bernabeu et al. 2009). Endoglin has been characterized an accessory receptor in TGF-β-ALK-1-dominated signaling in endothelial cells (Blanco et al. 2005). Importantly, endoglin regulates endothelial proliferation and migration (Jin et al. 2017; Sugden et al. 2017). In light of the central protumorigenic role attributed to TGF-β, the present study focused on the potential biological role of endoglin in glioblastoma.

## Materials and methods

### Cell lines, reagents, and transfections

The long-term glioblastoma cell lines (LTC) LN-18, LN-428, LN-319, A172, U87MG, T98G, LN-308, and LN-229) (Weller et al. 1998) were cultured in DMEM supplemented with 10% fetal bovine serum and 1% glutamine (Thermo Fisher Scientific, Waltham, MA, USA). U87MG has become a controversial cell line for lack of reproducible identity across laboratories, but is the best known glioma model and has therefore been traditionally included in our studies. The glioma-initiating cell (GIC) lines T-325, T-269, ZH-161, S-24, and ZH-305 (Le Rhun et al. 2019) were maintained in phenol-red free neurobasal medium with 2% B-27 without vitamin A (20 μl/ml), Glutamax (10 μl/ml, Invitrogen, Basel, Switzerland), fibroblast growth factor (FGF)-2, and epidermal growth factor (EGF) (20 ng/ml each; Peprotech, London, UK). The human brain endothelial cell line hCMEC (Weksler et al. 2005) was cultured as described (Seystahl et al. 2015). Human endothelial CD31 + cells isolated from freshly dissected glioblastoma samples were isolated and cultured as described (Krishnan et al. 2015). All cells were sent for short tandem repeat analysis (DSMZ, Braunschweig, Germany) and regularly tested for mycoplasma contamination, last in 2016 (cell culture experiments reported herein were concluded in 2017). Recombinant human (rh) TGF-β1 and BMP-10 were obtained from R&D Systems (Minneapolis, MN, USA). Transfection was performed using the Lipofectamine RNAiMAX reagent (Thermo Fisher Scientific). For transient gene silencing, siRNA pools provided by Dharmacon (Lafayette, CO, USA) were used at 100 nM final concentration.

### Patients and specimens

The specimens and patient data were retrieved and analyzed based on project outlines approved by the appropriate institutional review board (Kantonale Ethikkommission Zürich, Switzerland, KEK-ZH-Nr./BASCE-Nr. 2016-00456).

### Real-time (RT-PCR)

Real-time PCR (RT-PCR) was performed as described (Seystahl et.al. 2015) and calculated with the Δ threshold cycle (Ct) method for relative quantification. *ADP-ribosylation factor 1* (Arf1) was used as a housekeeping gene. Primer sequences were Arf forward 5′-TCC CAC ACA GTG AAG CTG ATG-3′, reverse 5′-GAC CAC GAT CCT CTA CAA GC-3′; endoglin forward 5′-TCA ACA TGG ACA GCC TCT CTT TC-3′, reverse 5′-GAC ACT CTG ACC TGC ACA AAG C-3′; CD31 forward 5′-TTT GGA CCA AGC AGA AGG CT-3′, reverse 5′-TTG GCC GCA ATG ATC AAG AGA-3′.

### Immunoblot analyses

Immunoblot analysis was performed as described (Tritschler et al. 2009). Primary antibodies were anti-endoglin/anti-CD105 antibody (ab137389, Abcam, Cambridge, UK), anti-Smad2 (3122S), anti-pSmad2 (3108S), anti-Smad5 (9517S), anti-pSmad1/5 (9516S), anti p44/42 MAPK (ERK1/2), anti-pp44/42 MAPK (ERK1/2) (Thr202, Tyr204), anti-AKT, anti-pAKT (Ser473), anti-pAKT (Thr308) (all Cell Signaling, Leiden, Netherlands), and anti-Actin (sc-1616, Santa Cruz Biotechnology, Inc., Dallas, TX, USA). Visualization of protein bands was accomplished using horseradish peroxidase (HRP)-coupled secondary antibodies (Santa Cruz Biotechnology) and enhanced chemiluminescence (Pierce/Thermo Fisher, Madison, WI, USA). Cell supernatants were collected from LTC at confluency, from GIC seeded in suspensions of 10^6^/ml and from endothelial cell lines isolated from freshly dissected glioblastoma samples seeded at 2.6 × 10^4^ cells/cm^2^ after 2 days. Cellular debris was removed by centrifugation. For LTC and GIC, results were expressed corresponding to the cell number at time of supernatant collection. For endothelial cell lines isolated from freshly dissected glioblastoma samples, supernatants were concentrated using Amicon® Ultra-4 centrifugal filter (Millipore Corporation) and results were expressed corresponding to the supernatant protein concentration measured using the Bradford Assay (Bio-Rad Laboratories, Berkeley, CA, USA).

### Flow cytometry

For the detection of endoglin in human glioma cell lines, we used anti-CD105-APC (BioLegend, San Diego, CA, USA) and an isotype-matched control antibody from eBioscience (San Diego, CA, USA). Specific fluorescence indexes (SFI) were calculated by dividing mean fluorescence obtained with the specific antibody by mean fluorescence obtained with isotype control antibody.

### Immunohistochemistry

Immunohistochemical stainings for endoglin were performed on a panel of 52 newly diagnosed and 10 recurrent glioblastoma specimens as previously described; data on 45 of these patients have been published previously (Frei et al. 2015). The paraffin sections were incubated over night with endoglin/anti-CD105 antibody (ab137389, Abcam, Cambridge, UK, concentration 1 mg/ml). Histofine simple stain Max PO (R) anti-rabbit (414351F, Cosmo Bio, Carlsbad, CA) was used as secondary antibody and incubated for 30 min at room temperature. The antigen antibody conjugates were detected by staining with diaminobenzidine (Dako, Glostrup, Denmark). The nuclei were stained using hematoxylin and dehydrated before mounting onto coverslips using Eukitt mounting medium (Sigma-Aldrich). Four representative images from the region of highest Ki67 staining were taken. The immunostaining was scored using the histo-score (H-score) (Goulding et al. 1995), ranging from 0 to 300 and calculated as the percentage of weakly stained cells plus the percentage of moderately stained cells multiplied by two plus the percentage of strongly stained cells multiplied by three.

### Single-cell real-time polymerase chain reaction (scRT-PCR) of reverse transcribed RNA

Single-cell real-time polymerase chain reaction (scRT-PCR) was performed on a set of six glioblastoma tissues as previously described (Ventura et al. 2018).

### Interrogations from The Cancer Genome Atlas (TCGA) network

Survival analyses were performed using the Kaplan-Meier analysis module of the R2 microarray analysis and visualization platform (https://r2.amc.nl). Survival data were obtained from the glioblastoma data set of TCGA network with the R2 internal identifier named ps_avgpres_broadgbm540_u133a. The gene expression data in this database were collected using Affymetrix gene chips. The query was based on the reporter with the highest mean geometric intensity for the target gene. The Affymetrix probesets ID used were as follows: ENG_201809_s_at for endoglin, CA9_205199_at for carbonic anhydrase IX (CAIX), VEGFA_210512_s_at for vascular endothelial growth factor A (VEGFA), FLT1_222033_s_at for vascular endothelial growth factor receptor (VEGFR) 1, and KDR_203934_at for VEGFR2. For survival analyses, different cut-offs were defined to segregate glioblastoma patients into two groups with high or low expression of the target gene: specifically, cut-offs were defined by the median expression level and the highest association with survival. For correlation analyses, the gene expression data were exported into GraphPadPrism Version 8 (San Diego, CA).

### Statistical analysis

Data were derived from at least two independent experiments with similar results and results of representative experiments are shown. Means, standard error of the mean (SEM), correlation (*r* = Pearson’s coefficient), linear regression, survival curves (Kaplan-Meier method with log-rank test), and statistical significance (using two-sided unpaired Student’s *t* test or one sample *t* test) were calculated using the software of GraphPad Prism Version 8 (San Diego, CA) and IBM Statistics, Version 25 (SPSS). A *p* value of *p* = 0.05 was considered to be statistically significant. Statistical analysis of patient data was performed as described (Frei et al. 2015).

## Results

### Endoglin expression in human glioblastoma in vivo

**Fig. 1:**
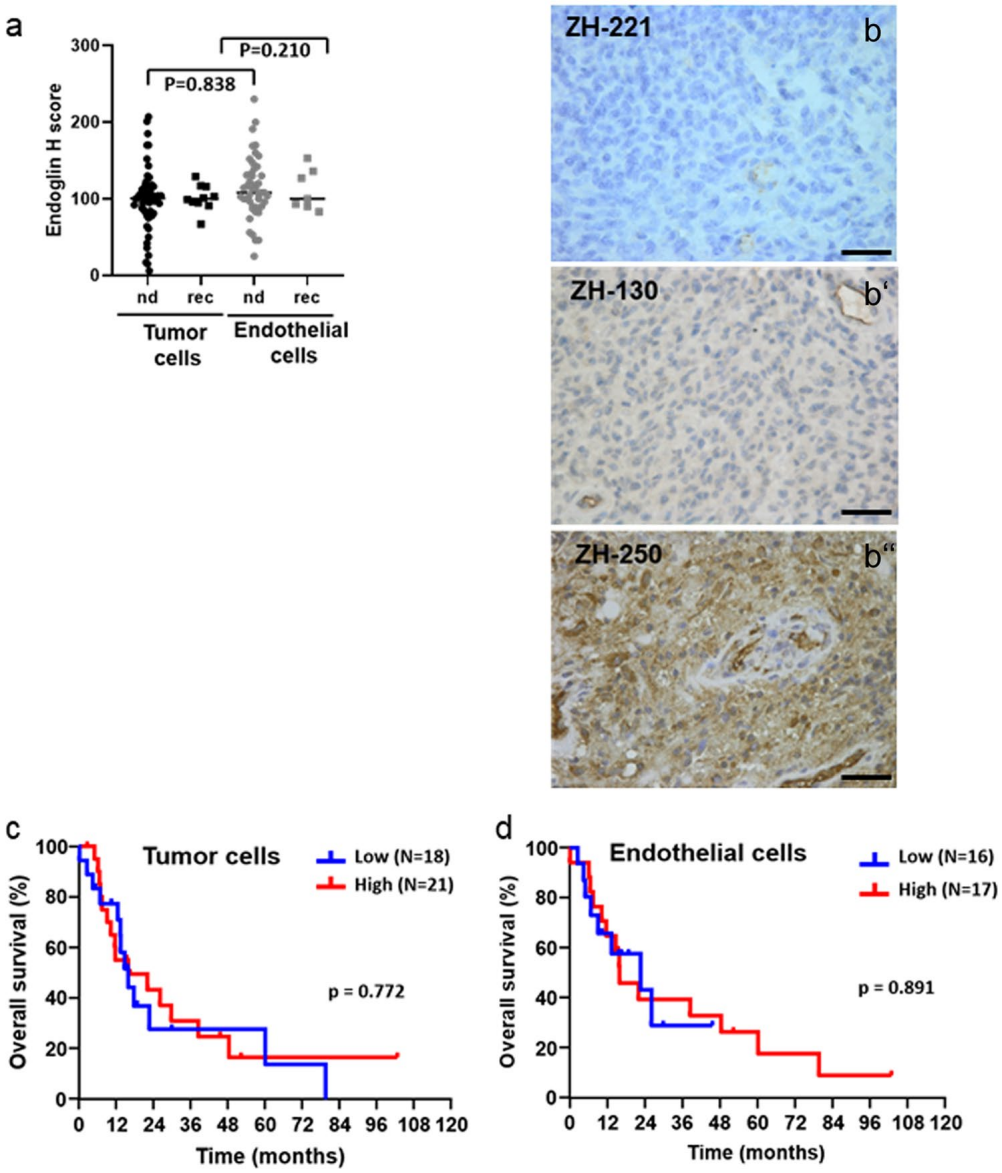
Endoglin is expressed in human glioblastoma tissue in vivo*.* (**a**) Glioblastoma tissue samples from 52 newly diagnosed (nd) and 10 recurrent (rec) glioblastoma patients were stained for endoglin by immunohistochemistry and manually analyzed for expression in tumor versus endothelial cells using the H-score method. Every single tissue sample is represented by a dot. (**b, b’, b’’**) Representative photomicrographs of stainings for endoglin (score < 50 in b, score 50–150 in b’, score > 150 in b’’) (size bar in the right corner: 50 µm). The ‘ZH’ headings denote codes for individual patients. (**c, d**) Kaplan-Meier survival curves are shown for IDH wild-type glioblastoma patients stratified by endoglin expression levels; the median expression level (median H-score) was used as cut-off to define patients with high (red) or low expression (blue). Data are shown for the expression in tumor cells (**c**) or endothelial compartment (**d**). The log-rank test was used for comparison

Protein levels of endoglin were determined by immunohistochemistry in 52 newly diagnosed and 10 recurrent glioblastoma tissue samples. Patient characteristics are summarized in Table 1. Manual analysis was performed separately for endothelial versus tumor cells based on morphological criteria using the H-score method. The median H-score for newly diagnosed glioblastoma was 100.5 (95% confidence interval (CI) 94–105) in tumor cells and 108.0 (95% CI 100–122) in endothelial cells and for recurrent glioblastoma 100.0 (95% CI 91–117) in tumor cells and 100.0 (95% CI 83–153) in endothelial cells. Immunohistochemistry analysis did not show differences of H-scores for endoglin protein levels between endothelial and tumor cell fractions when looking at the samples as cohorts (newly diagnosed *p* = 0.838; recurrent *p* = 0.210) (Fig. 1a). Representative sections of staining with an H-score < 50 (Fig. 1b), score 50–150 (Fig. 1b'), and score > 150 (Fig. 1b'') are shown. Tissue sections of newly diagnosed and recurrent tumor of the same patient for two patients were compared and showed no change of endoglin levels upon recurrence (Fig. S1). Correlation analysis was performed in the group of newly diagnosed glioblastoma (*N* = 52) or in the group of recurrent glioblastoma (*N* = 10). There was significant correlation between endoglin expression in the tumor cells and endoglin expression in the endothelial cells (Fig. S2, Table 2). Moreover, a positive correlation was seen between endoglin and TGF-β1 protein (*r* = 0.453, *p* = 0.006) and PAI-1 protein (*r* = 0.350, *p* = 0.036) in tumor cells of newly diagnosed glioblastoma, as well as a positive significant correlation of endoglin and pSmad1/5 levels in the tumor cells of the newly diagnosed and recurrent patients (*r* = 0.479, *p* = 0.004 and *r* = 0.885, *p* = 0.019, respectively) (Table 2). Survival data were available for 39 patients with newly diagnosed IDH wild-type tumors and information on endoglin levels in tumor cells and for 33 patients with known endoglin expression in the endothelial compartment. Median overall survival (OS) was compared between patients with low and high levels of endoglin in the tumor cells and the endothelial cells, respectively. The cut-off was defined by the median H-score. No relevant differences were observed between low and high endoglin-expressing tumors, neither for the analysis of tumor cell expression (*p* = 0.772) (Fig. 1c), nor for endothelial expression (*p* = 0.891) (Fig. 1d). Analyzing survival of glioblastoma patients of the TCGA database also showed no association of endoglin expression with survival when median expression level defined the cut-off for dividing glioblastoma patients into those with high or low expression (Fig. S3a). Still, enhanced expression of endoglin mRNA was associated with inferior survival when the expression cut-off was defined individually for the statistically ideal cut-off (Fig. S3b).

**Table 1:** Patient characteristics

	Newly diagnosed glioblastoma *N* = 52^a^	Recurrent glioblastoma *N* = 10^a^
Age, years		
Median (range)	62 (32–79)	49 (20–79)
Gender, *N* (%)		
Female	22 (42.3)	1 (10.0)
Male	30 (57.7)	9 (90.0)
KPS^b^, *N* (%)		
< 70%	13 (25.5)	1 (11.1)
70–80%	29 (56.9)	5 (55.6)
90–100%	9 (17.6)	3 (33.3)
No data	1 (-)	1 (-)
Extent of resection, *N* (%)		
Biopsy	2 (3.9)	0 (0)
Incomplete (< 99%)	35 (68.6)	5 (55.6)
Gross total	14 (27.5)	4 (44.4)
No data	1 (-)	1 (-)
First-line therapy, *N* (%)		
No therapy	1 (2.0)	0 (0)
Radiotherapy alone	13 (26.0)	2 (22.2)
Alkylating chemotherapy alone	2 (4.0)	0 (0)
Radiotherapy plus chemotherapy	34 (68.0)	7 (77.8)
No data	2 (-)	1 (-)
IDH mutation status		
IDH wild-type	39 (97.5)	2 (66.7)
IDH mutant	1 (2.5)	1 (33.3)
No data	12 (-)	6 (-)

*KPS* Karnofsky performance score, *IDH* isocitrate dehydrogenase

^a^*N* = 4 patients had tissue at time of diagnosis and at time of recurrence

^b^At time of diagnosis or recurrence (postoperatively), respectively

**Table 2:** Correlation analyses

	Endoglin (H-score)			
	Newly diagnosed		Recurrent	
	Tumor cells	Endothelial cells	Tumor cells	Endothelial cells
TGF-β1 (mRNA)	*r* = 0.005 *p* = 0.976	*r* = 0.019 *p* = 0.920	*r* = -0.522 *p* = 0.185	*r* = -0.423 *p* = 0.404
TGF-β2 (mRNA)	*r* = -0.092 *p* = 0.592	*r* = 0.051 *p* = 0.791	*r* = -0–240 *p* = 0.567	*r* = -0.158 *p* = 0.764
TGF-β3 (mRNA)	*r* = 0.014 *p* = 0.935	*r* = 0.071 *p* = 0.709	*r* = -0.546 *p* = 0.161	*r* = -0.426 *p* = 0.400
TGF-β1 (H-score)	*r* = 0.453 *p* = 0.006**	*r* = 0.275 *p* = 0.141	*r* = 0.466 *p* = 0.292	*r* = 0.371 *p* = 0.538
TGF-β2 (H-score)	*r* = -0.028 *p* = 0.871	*r* = 0.001 *p* = 0.998	*r* = 0.598 *p* = 0.157	*r* = 0.749 *p* = 0.145
TGF-β3 (H-score)	*r* = 0.259 *p* = 0.128	*r* = 0.035 *p* = 0.856	*r* = 0.369 *p* = 0.416	*r* = 0.467 *p* = 0.428
pSmad2 (H-score)	*r* = 0.073 *p* = 0.673	*r* = 0.044 *p* = 0.819	*r* = 0.459 *p* = 0.300	*r* = 0.891 *p* = 0.043*
pSmad1/5 (H-score)	*r* = 0.479 *p* = 0.004**	*r* = 0.615 *p* < 0.001***	*r* = 0.885 *p* = 0.019*	*r* = -0.530 *p* = 0.470
PDGF-B (mRNA)	*r* = 0.037 *p* = 0.831	*r* = -0.033 *p* = 0.864	*r* = -0.196 *p* = 0.642	*r* = -0.151 *p* = 0.775
PAI-1 (mRNA)	*r* = 0.012 *p* = 0.944	*r* = 0.062 *p* = 0.746	*r* = -0.342 *p* = 0.408	*r* = -0.290 *p* = 0.578
PAI-1 (H-score)	*r* = 0.350 *p* = 0.036*	*r* = 0.325 *p* = 0.080	*r* = -0.016 *p* = 0.974	*r* = -0.236 *p* = 0.702
Ki-67 (protein expression)	*r* = -0.347 *p* = 0.036*	*r* = -0.353 *p* = 0.066	*r* = -0.601 *p* = 0.154	*r* = -0.350 *p* = 0.563
Endoglin endothelial cells (H-score)	*r* = 0.751 *p* < 0.001***		*r* = 0.954 *p* < 0.001***	

Statistical significances of *p* < 0.05 (*), *p* < 0.01 (**), and *p* < 0.001 (***) were determined using the Pearson’s correlation test (*r*, correlation coefficient)

**Fig. 2:**
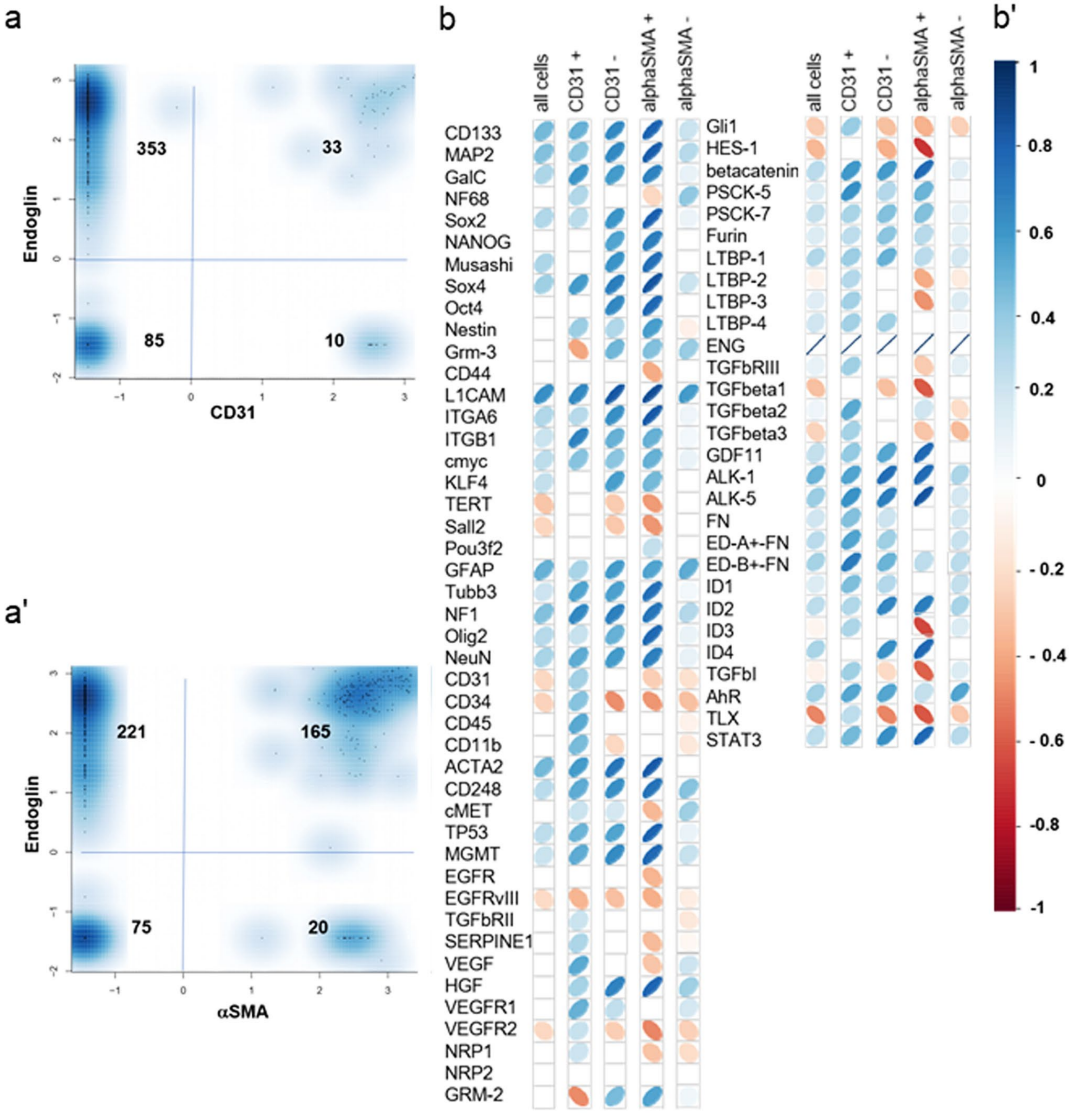
Endoglin expression on single-cell level in freshly dissociated human glioblastoma tissue. (**a, a’**) Single-cell RT-PCR was performed and relative mRNA gene expression of endoglin in individual cells derived from freshly dissociated human glioblastoma is depicted. Data from 437 individual cells (pooled from 6 tumors to ensure statistical reliability) were divided into CD31+ and CD31− cells (**a**), or into $\alpha$SMA+ and $\alpha$SMA− cells (**a’**). (**b**) Correlation matrix of endoglin expression and other genes as indicated in the figure in all cells pooled, in CD31+ and CD31- cells and in αSMA+ and αSMA− cells analyzed separately. (**b’**) Correlation coefficients are color-encoded as indicated in the bar and only indicated in the matrix in (**b**) in case of significance

There are reports about the induction of endoglin by hypoxia in human endothelial cells (Li et al. 2003). Therefore, we investigated the association of *endoglin* with the hypoxia-responsive genes *CAIX*, *VEGFA*, *VEGFR1*, and *VEGFR2* in glioblastoma samples from the TCGA database. The expression level of endoglin was positively correlated with the expression level of each of these genes (Fig. S4). However, we did not detect an upregulation of endoglin by flow cytometry in a human LTC (LN-18) or GIC (ZH-161) glioma cell line after culturing under hypoxia compared with normoxia (Fig. S5). To analyze expression of endoglin on single-cell level, we performed single-cell real-time polymerase chain reaction (scRT-PCR) in cells from six freshly dissociated human glioblastoma tissues using CD31 and $\alpha$SMA as markers of endothelial cells and pericytes/VSMC, respectively. Of the 481 single cells analyzed, 43 cells were CD31-positive and 185 were $\alpha$SMA-positive (Fig. S6). Endoglin mRNA was expressed in most cells with 77% of the CD31-positive, 80% of the CD31-negative cells (Fig. 2a), 89% of the $\alpha$SMA-positive, and 75% of the $\alpha$SMA-negative cells being positive (Fig. 2a). Correlation analysis of all 481 cells of the six glioblastoma patients on single-cell level revealed correlations of endoglin expression (*p* < 0.001) with the stem cell marker L1 cell adhesion molecule (L1CAM) (*r* = 0.297), the astrocyte marker glial fibrillary acidic protein (GFAP) (*r* = 0.283), the pericyte/vascular smooth muscle cell marker $\alpha$SMA (*r* = 0.256), the neuronal marker microtubule-associated protein (MAP) 2 (*r* = 0.256), neurofibromatosis (NF) 1 gene (*r* = 0.231), and ALK-1 (*r* = 0.240) (Fig. 2b).

### Endoglin expression in human glioblastoma in vitro

**Fig. 3:**
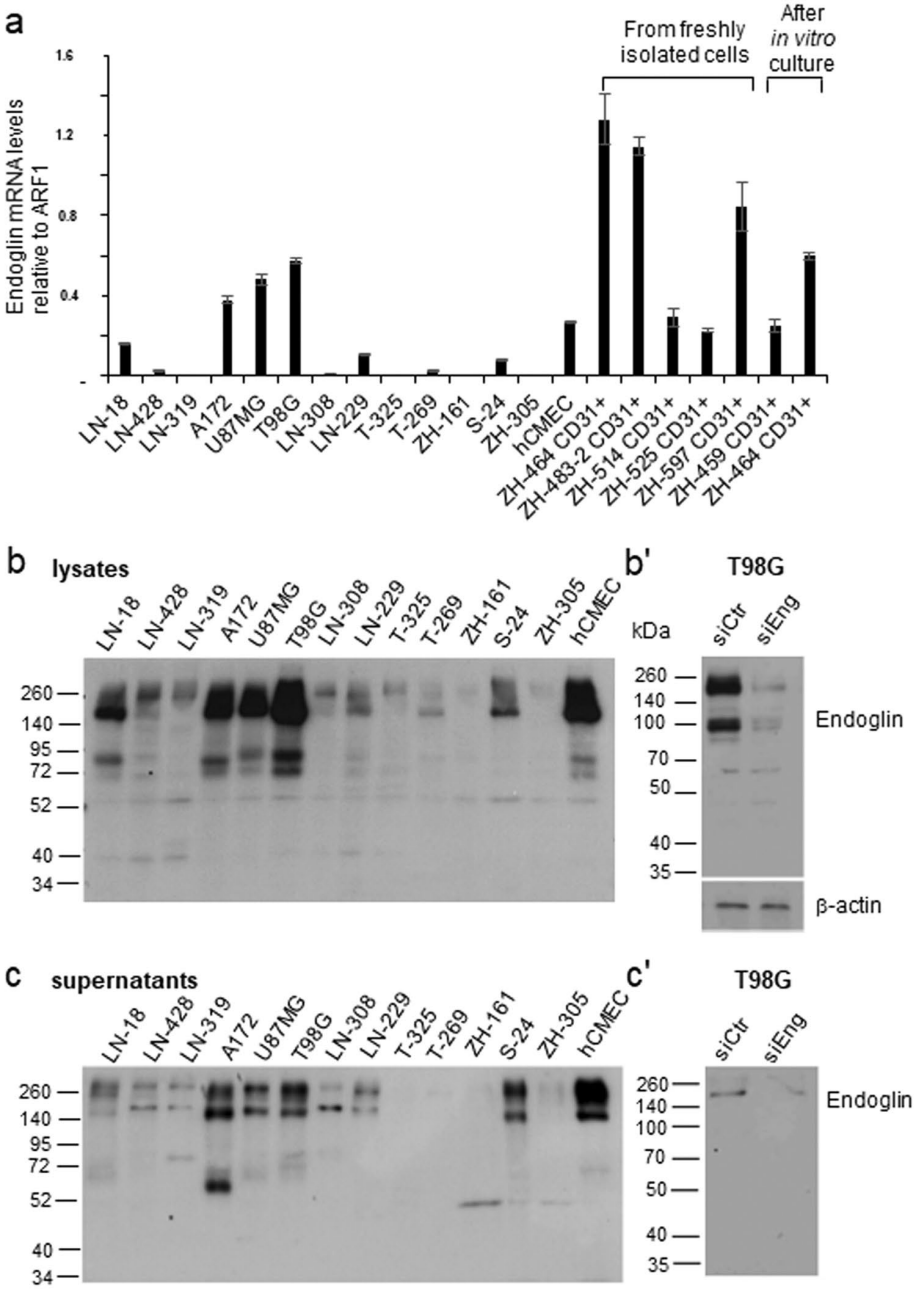
Endoglin expression in human glioma cells in vitro. (**a**) Endoglin mRNA expression in human LTC (LN-18, LN-428, LN-319, A172, U87MG, T98G, LN-308, LN-229), GIC (T-325, T-269, ZH-161, S-24, ZH-305), hCMEC, and CD31+ cells derived from freshly dissociated human glioblastoma (as indicated directly analyzed from freshly isolated cells or after in vitro culture) normalized to Arf1. (**b, b’, c, c’**) Human LTC, GIC, or hCMEC were incubated in serum-free medium for 48 h and whole cell lysates (**b, b’**) and concentrated supernatants (**c, c’**) were analyzed by immunoblot under nonreducing conditions. (**b’, c’**) T98G cells were transfected with a control siRNA or siRNA targeting endoglin. At 24 h, post-transfection cells were put in serum-free medium for 48 h. (**b’**) Whole cell lysates were generated and analyzed by immunoblot under nonreducing conditions; equal protein loading was confirmed by actin staining. (**c’**) The respective conditioned culture media was concentrated and analyzed for the levels of endoglin under nonreducing conditions

**Fig. 4:**
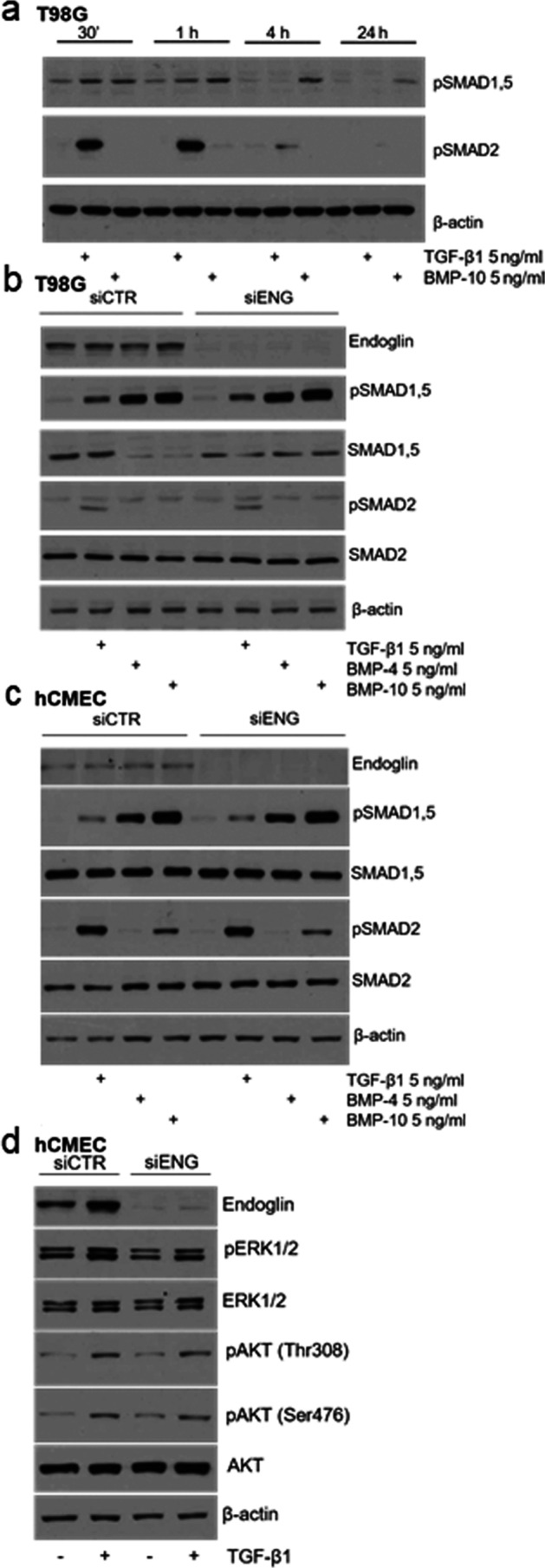
No major role for endoglin in regulating SMAD-dependent nor SMAD-independent TGF-β signaling. (**a**) T98G cells were treated with recombinant BMP-10 or TGF-β1 (5 ng/ml, respectively) for 30 min, 1 h, 4 h, and 24 h and the levels of phosphorylated SMAD1,5 and SMAD2 were determined by immunoblot. (**b**) T98G cells transfected with non-targeting control siRNA or siRNA targeting endoglin were treated with recombinant TGF-β1, BMP-4, or BMP-10 (5 ng/ml for 30 min, respectively). The levels of phosphorylated and total SMAD1,5 and SMAD2 were determined by immunoblot. (**c**) hCMEC cells transfected with non-targeting control siRNA or siRNA targeting endoglin were treated with recombinant TGF-β1, BMP-4, or BMP-10 (5 ng/ml for 30 min, respectively). (**d**) Levels of endoglin, pERK1/2, pAKT (Thr308), and pAKT (Ser476) and of total ERK1/2 and AKT were determined in the respective lysates by immunoblot

Next, endoglin mRNA expression was determined in a panel of 13 human glioma cell lines, 8 LTC and 5 GIC. Further, endoglin mRNA expression of a human cerebral microvascular endothelial cell line (hCMEC) and of CD31+ cells isolated from freshly dissociated human glioblastoma tissues was analyzed. Five of these ex vivo CD31+ endothelial cell cultures had been analyzed for endoglin mRNA directly after surgery and enrichment. Two endothelial cell lines (ZH-459 CD31+ cells and ZH-464 CD31+-positive cells) have been described before (Krishnan et al. 2015) and had been cultured for less than ten passages. Endoglin expression was variable among glioma cell lines, with the highest expression levels in three LTC, A172, U87MG, and T98G, which was comparable with the levels of hCMEC and serially passaged CD31+ cells isolated from glioblastoma tissues; endoglin mRNA expression in GIC was low (Fig. 3a). We analyzed the levels of endoglin in cell lysates (Fig. 3b) and in the conditioned medium (Fig. 3c), representing the levels of membrane-bound versus shed or soluble endoglin. Under non-reducing conditions, endoglin was detected in the cell lysates as two major bands with an apparent molecular mass between 140 and 260 kDa and as a double band of around 100 kDa (Fig. 3b). In the supernatants, endoglin was detected as two major bands between 140 and 260 kDa similar to the ones detected in cell lysates (Fig. 3c). These bands disappeared upon endoglin gene silencing in T98G cells in lysates and in supernatants (Fig. 3b, c). Since 180 kDa is the molecular mass reported for homodimeric endoglin, these bands are consistent with the dimeric and monomeric forms of the protein (Fig. 3b). Under reducing conditions endoglin was not detectable in the supernatants (data not shown). Overall, endoglin mRNA and protein levels and specifically endoglin levels in the supernatants and cell lysates roughly correlated, indicating constitutive shedding of endoglin. TGF-β1 (Cheifetz et al. 1992) and BMP-9 and BMP-10 (Castonguay et al. 2011) have been found to directly bind to the extracellular domain of endoglin whereas other TGF-β superfamily ligands may bind endoglin only in the presence of their respective type I and type II receptors (Barbara et al. 1999). To obtain further insight into the role of endoglin in the signal transduction of these ligands, we treated T98G cells with TGF-β1 or BMP-10 at different time points. TGF-β1 induced the phosphorylation of both Smad1,5 and Smad2 whereas BMP-10 increased the levels of pSMAD1,5 only (Fig. 4a). Since pSmad2 was induced by TGF-β1 after 30 min of stimulation (Fig. 4a) and stimulation decreased already after 1 h and disappeared after 4 h, further stimulation experiments were performed at 30 min. Endoglin gene silencing did not affect the basal levels of pSmad1,5 and pSmad2. Furthermore, upon stimulation with TGF-β1, BMP-4, or BMP-10, their levels remained unaltered in T98G glioma cells (Fig. 4b). In hCMEC cells, we confirmed a faint increase in constitutive pSmad1/5 levels, but no effect on pSmad2 levels upon endoglin gene silencing (Krishnan et al. 2015). There was no change in pSmad1/5 or pSmad2 levels upon stimulation with the TGF-β superfamily ligands TGF-β1, BMP-4, and BMP-10 in the endoglin-depleted cells compared with the sicontrol cells (Fig. 4c). Likewise, with regard to noncanonical Smad-independent TGF-β signaling, endoglin gene silencing did not affect the levels of phosphorylated ERK1/2 and AKT at basal levels or after stimulation with TGF-β superfamily ligands (Fig. 4d).

## Discussion/conclusion

Glioblastoma are highly vascular tumors and the identification of alternative angiogenic pathways including the possibility of their therapeutic targeting represents a challenge of outstanding importance in clinical neuro-oncology. Our previous studies indicated that TGF-β-dependent signal transduction may play an important role in angiogenesis in glioma, including crosstalk with VEGF signaling (Mangani et al. 2016; Seystahl et al. 2015). Various therapeutic approaches targeting glioma-derived TGF-β have been proposed, but only few have been tested in human glioblastoma patients. The most advanced strategy is to block TGF-βRI kinase activity by small molecules. Yet, neither monotherapy with LY2157299 nor therapy with LY2157299 in combination with lomustine prolonged overall survival in patients with recurrent glioblastoma (Brandes et al. 2016). Early programs aiming at inhibiting TGF-β bioactivity focusing on the local administration of oligonucleotides reducing expression of the gene were disappointing (Bogdahn et al. 2011), but novel more powerful agents have recently been developed (Papachristodoulou et al. 2019).

Regulation of TGF-β signaling is dependent on multiple factors including the presence of accessory receptors such as TGF-βRIII and endoglin as functional components of the membrane TGF-β receptor complex. Specifically, endoglin has been reported to be predominantly expressed on endothelial cells and has been attributed an important regulator in ALK-1/Smad1/5/8-dependent signaling. The TGF-β/Smad2 and the TGF-β/Smad1/5/8 pathway have been demonstrated to have opposite functional effects in endothelial cell models (Goumans et al. 2003). An association of overall survival and number of blood vessels positively stained for endoglin has been demonstrated in pediatric high-grade gliomas (Smith et al. 2012). Here, we show that endoglin protein levels do not differ comparing glioblastoma vasculature and tumor tissue (Fig. 1a, b). Endoglin staining was not restricted to tumor vasculature (representative pictures in Fig. 1b), but tumor stroma also had a positive endoglin staining similar to results for other tumor entities such as Ewing sarcoma and melanoma (Pardali et al. 2011). This is in line with the observation that glioma cells express endothelial markers (Soda et al. 2011). A high density of endoglin-expressing microvessels has been associated with a poor prognosis in patients with glioblastoma (Yao et al. 2005). In the TCGA database, we only identified a trend for inferior survival in patients with glioblastoma with high expression of endoglin (Fig. 1c, d), but we did not investigate endoglin-expressing microvascular density in our cohort. By single-cell expression analysis from freshly dissociated human glioblastoma samples, we confirmed the results of the protein staining with similar levels of endoglin mRNA in the vascular compartment and the tumor cells (Fig. 2a). Correlation analyses revealed correlations of endoglin mRNA with the stem cell markers L1CAM and CD133 and with ALK-1 (Fig. 2b). Endoglin has been reported to potentiate TGF-β/ALK-1 signaling (Blanco et al. 2005). Among glioma cell lines, endoglin mRNA expression levels were highly variable (Fig. 3a). We observed endoglin protein in cellular lysates and in cell culture media with similar apparent molecular size as a double band between 140 and 240 kDa. This similar size of membrane bound and soluble endoglin accounts for endoglin shedding close to the transmembrane domain (Fig. 3b, c). Indeed, for colorectal cancer cells, it has been reported that MMP-14 cleaves endoglin at position 586–587, releasing a fragment close to the size of the complete ECD with the transmembrane domain spanning 71 amino acids only (Hawinkels et al. 2010). Specifically, in the context of epithelial cells, endoglin has been described as an important cofactor in the ALK-1/Smad1 pathway, with endoglin and ALK-1 interacting physically (Blanco et al. 2005). In contrast, in our glioma and endothelial cell models, endoglin gene silencing did not strongly influence constitutive or ligand-evoked Smad-dependent signaling or non-canonical TGF-β superfamily-dependent signaling (Fig. 4). For the glioma cell lines, an explanation may be the absence of TGF-β/ALK-1 signaling since ALK-1 protein was not detected (Seystahl et al. 2015). Further investigations may be necessary to identify other signaling pathways with involvement of endoglin. Interestingly, recently, endoglin-dependent Wnt-mediated transcriptional changes have been identified in progenitor cells of zebrafish and mouse models (Baik et al. 2016).

Hypoxia has been reported as a stimulus for endoglin induction in human endothelial cells and we identified an association of endoglin with hypoxia-responsive genes in glioblastoma patient samples from the TCGA database (Fig. S4). However, we did not detect an upregulation of endoglin in glioma cell lines after culturing under hypoxia (Fig. S5). Thus, the association of endoglin expression with other hypoxia-responsive genes in human glioblastoma samples may not be caused by a direct hypoxia-mediated upregulation of endoglin on glioma cells, but rather by more complex mechanisms on the tumor microenvironment such as endothelial cell proliferation (Miller et al. 1999).

Despite the limited knowledge on the potential impact of endoglin modulation in glioblastoma, it is noteworthy that endoglin-targeted cancer therapy has been explored in patients with recurrent glioblastoma (ClinicalTrials.gov Identifier: NCT01648348). A more detailed understanding of endoglin’s specific impact in brain tumors should be obtained prior to further clinical trials of anti-endoglin therapy.

## Statement of ethics

The specimens and patient data were retrieved in accordance with the permission of the institutional review board and after obtaining informed consent. The analysis was performed according to the guidelines of the local ethics committees (Kantonale Ethikkommission Zürich, Switzerland, KEK-ZH-Nr./BASCE-Nr. 2016-00456).

## Conflict of interest

MW has received research grants from Abbvie, Adastra, Bristol Meyer Squibb (BMS), Dracen, Merck, Sharp & Dohme (MSD), Merck (EMD), Novocure, Piqur and Roche, and honoraria for lectures or advisory board participation or consulting from Abbvie, Basilea, Bristol Meyer Squibb (BMS), Celgene, Merck, Sharp & Dohme (MSD), Merck (EMD), Novocure, Orbus, Roche, and Tocagen. All other authors declare no conflict of interest.

## Electronic supplementary material

Below is the link to the electronic supplementary material.

Supplementary file1 (DOCX 1.1 MB)

## References

[CR1] Baik J, Magli A, Tahara N, Swanson SA, Koyano-Nakagawa N, Borges L, Stewart R, Garry DJ, Kawakami Y, Thomson JA, Perlingeiro RC (2016). Endoglin integrates BMP and Wnt signalling to induce haematopoiesis through JDP2. Nat Commun.

[CR2] Barbara NP, Wrana JL, Letarte M (1999). Endoglin is an accessory protein that interacts with the signaling receptor complex of multiple members of the transforming growth factor-beta superfamily. J Biol Chem.

[CR3] Bernabeu C, Lopez-Novoa JM, Quintanilla M (2009). The emerging role of TGF-beta superfamily coreceptors in cancer. Biochim Biophys Acta.

[CR4] Blanco FJ, Santibanez JF, Guerrero-Esteo M, Langa C, Vary CP, Bernabeu C (2005). Interaction and functional interplay between endoglin and ALK-1, two components of the endothelial transforming growth factor-beta receptor complex. J Cell Physiol.

[CR5] Bogdahn U, Hau P, Stockhammer G, Venkataramana NK, Mahapatra AK, Suri A, Balasubramaniam A, Nair S, Oliushine V, Parfenov V, Poverennova I, Zaaroor M, Jachimczak P, Ludwig S, Schmaus S, Heinrichs H, Schlingensiepen KH, Trabedersen Glioma Study G (2011). Targeted therapy for high-grade glioma with the TGF-beta2 inhibitor trabedersen: results of a randomized and controlled phase IIb study. Neuro Oncol.

[CR6] Brandes AA, Carpentier AF, Kesari S, Sepulveda-Sanchez JM, Wheeler HR, Chinot O, Cher L, Steinbach JP, Capper D, Specenier P, Rodon J, Cleverly A, Smith C, Gueorguieva I, Miles C, Guba SC, Desaiah D, Lahn MM, Wick W (2016). A phase II randomized study of galunisertib monotherapy or galunisertib plus lomustine compared with lomustine monotherapy in patients with recurrent glioblastoma. Neuro Oncol.

[CR7] Castonguay R, Werner ED, Matthews RG, Presman E, Mulivor AW, Solban N, Sako D, Pearsall RS, Underwood KW, Seehra J, Kumar R, Grinberg AV (2011). Soluble endoglin specifically binds bone morphogenetic proteins 9 and 10 via its orphan domain, inhibits blood vessel formation, and suppresses tumor growth. J Biol Chem.

[CR8] Cheifetz S, Bellon T, Cales C, Vera S, Bernabeu C, Massague J, Letarte M (1992). Endoglin is a component of the transforming growth factor-beta receptor system in human endothelial cells. J Biol Chem.

[CR9] Chinot OL, Wick W, Cloughesy T (2014). Bevacizumab for newly diagnosed glioblastoma. N Engl J Med.

[CR10] Das S, Marsden PA (2013). Angiogenesis in glioblastoma. N Engl J Med.

[CR11] Frei K, Gramatzki D, Tritschler I, Schroeder JJ, Espinoza L, Rushing EJ, Weller M (2015). Transforming growth factor-beta pathway activity in glioblastoma. Oncotarget.

[CR12] Gilbert MR, Dignam JJ, Armstrong TS, Wefel JS, Blumenthal DT, Vogelbaum MA, Colman H, Chakravarti A, Pugh S, Won M, Jeraj R, Brown PD, Jaeckle KA, Schiff D, Stieber VW, Brachman DG, Werner-Wasik M, Tremont-Lukats IW, Sulman EP, Aldape KD, Curran WJ, Mehta MP (2014). A randomized trial of bevacizumab for newly diagnosed glioblastoma. The New England journal of medicine.

[CR13] Goulding H, Pinder S, Cannon P, Pearson D, Nicholson R, Snead D, Bell J, Elston CW, Robertson JF, Blamey RW (1995). A new immunohistochemical antibody for the assessment of estrogen receptor status on routine formalin-fixed tissue samples. Hum Pathol.

[CR14] Goumans MJ, Valdimarsdottir G, Itoh S, Lebrin F, Larsson J, Mummery C, Karlsson S, ten Dijke P (2003). Activin receptor-like kinase (ALK)1 is an antagonistic mediator of lateral TGFbeta/ALK5 signaling. Mol Cell.

[CR15] Gramatzki D, Roth P, Rushing EJ, Weller J, Andratschke N, Hofer S, Korol D, Regli L, Pangalu A, Pless M, Oberle J, Bernays R, Moch H, Rohrmann S, Weller M (2018). Bevacizumab may improve quality of life, but not overall survival in glioblastoma: an epidemiological study. Ann Oncol.

[CR16] Hawinkels LJ, Kuiper P, Wiercinska E, Verspaget HW, Liu Z, Pardali E, Sier CF, ten Dijke P (2010). Matrix metalloproteinase-14 (MT1-MMP)-mediated endoglin shedding inhibits tumor angiogenesis. Cancer Res.

[CR17] Jin Y, Muhl L, Burmakin M, Wang Y, Duchez AC, Betsholtz C, Arthur HM, Jakobsson L (2017). Endoglin prevents vascular malformation by regulating flow-induced cell migration and specification through VEGFR2 signalling. Nat Cell Biol.

[CR18] Krishnan S, Szabo E, Burghardt I, Frei K, Tabatabai G, Weller M (2015) Modulation of cerebral endothelial cell function by TGF-beta in glioblastoma: VEGF-dependent angiogenesis versus endothelial mesenchymal transition. Oncotarget 10.18632/oncotarget.4310 PMC4673177 26090865

[CR19] Le Rhun E, von Achenbach C, Lohmann B, Silginer M, Schneider H, Meetze K, Szabo E, Weller M (2019). Profound, durable and MGMT-independent sensitivity of glioblastoma cells to cyclin-dependent kinase inhibition. Int J Cancer.

[CR20] Li C, Issa R, Kumar P, Hampson IN, Lopez-Novoa JM, Bernabeu C, Kumar S (2003). CD105 prevents apoptosis in hypoxic endothelial cells. J Cell Sci.

[CR21] Mangani D, Weller M, Seyed Sadr E, Willscher E, Seystahl K, Reifenberger G, Tabatabai G, Binder H, Schneider H (2016). Limited role for transforming growth factor-beta pathway activation-mediated escape from VEGF inhibition in murine glioma models. Neuro Oncol.

[CR22] Miller DW, Graulich W, Karges B, Stahl S, Ernst M, Ramaswamy A, Sedlacek HH, Müller R, Adamkiewicz J (1999). Elevated expression of endoglin, a component of the TGF-beta-receptor complex, correlates with proliferation of tumor endothelial cells. Int J Cancer.

[CR23] Miyazawa K, Shinozaki M, Hara T, Furuya T, Miyazono K (2002). Two major Smad pathways in TGF-beta superfamily signalling. Genes Cells.

[CR24] Moustakas A, Heldin CH (2005). Non-Smad TGF-beta signals. J Cell Sci.

[CR25] Papachristodoulou A, Silginer M, Weller M, Schneider H, Hasenbach K, Janicot M, Roth P (2019). Therapeutic targeting of TGFbeta ligands in glioblastoma using novel antisense oligonucleotides reduces the growth of experimental gliomas. Clin Cancer Res.

[CR26] Pardali E, van der Schaft DW, Wiercinska E, Gorter A, Hogendoorn PC, Griffioen AW, ten Dijke P (2011). Critical role of endoglin in tumor cell plasticity of Ewing sarcoma and melanoma. Oncogene.

[CR27] Schmierer B, Hill CS (2007). TGFbeta-SMAD signal transduction: molecular specificity and functional flexibility. Nat Rev Mol Cell Biol.

[CR28] Seystahl K, Tritschler I, Szabo E, Tabatabai G, Weller M (2015). Differential regulation of TGF-beta-induced, ALK-5-mediated VEGF release by SMAD2/3 versus SMAD1/5/8 signaling in glioblastoma. Neuro Oncol.

[CR29] Smith SJ, Tilly H, Ward JH, Macarthur DC, Lowe J, Coyle B, Grundy RG (2012). CD105 (Endoglin) exerts prognostic effects via its role in the microvascular niche of paediatric high grade glioma. Acta Neuropathol.

[CR30] Soda Y, Marumoto T, Friedmann-Morvinski D, Soda M, Liu F, Michiue H, Pastorino S, Yang M, Hoffman RM, Kesari S, Verma IM (2011). Transdifferentiation of glioblastoma cells into vascular endothelial cells. Proc Natl Acad Sci U S A.

[CR31] Sugden WW, Meissner R, Aegerter-Wilmsen T, Tsaryk R, Leonard EV, Bussmann J, Hamm MJ, Herzog W, Jin Y, Jakobsson L, Denz C, Siekmann AF (2017). Endoglin controls blood vessel diameter through endothelial cell shape changes in response to haemodynamic cues. Nat Cell Biol.

[CR32] ten Dijke P, Arthur HM (2007). Extracellular control of TGFbeta signalling in vascular development and disease. Nat Rev Mol Cell Biol.

[CR33] Tritschler I, Gramatzki D, Capper D, Mittelbronn M, Meyermann R, Saharinen J, Wick W, Keski-Oja J, Weller M (2009). Modulation of TGF-beta activity by latent TGF-beta-binding protein 1 in human malignant glioma cells. Int J Cancer.

[CR34] Venkatesha S, Toporsian M, Lam C, Hanai J, Mammoto T, Kim YM, Bdolah Y, Lim KH, Yuan HT, Libermann TA, Stillman IE, Roberts D, D'Amore PA, Epstein FH, Sellke FW, Romero R, Sukhatme VP, Letarte M, Karumanchi SA (2006). Soluble endoglin contributes to the pathogenesis of preeclampsia. Nat Med.

[CR35] Ventura E, Weller M, Macnair W, Eschbach K, Beisel C, Cordazzo C, Claassen M, Zardi L, Burghardt I (2018) TGF-beta induces oncofetal fibronectin that, in turn, modulates TGF-beta superfamily signaling in endothelial cells. J Cell Sci 131:10.1242/jcs.209619 29158223

[CR36] Wang J, Cazzato E, Ladewig E, Frattini V, Rosenbloom DI, Zairis S, Abate F, Liu Z, Elliott O, Shin YJ, Lee JK, Lee IH, Park WY, Eoli M, Blumberg AJ, Lasorella A, Nam DH, Finocchiaro G, Iavarone A, Rabadan R (2016). Clonal evolution of glioblastoma under therapy. Nat Genet.

[CR37] Weksler BB, Subileau EA, Perriere N, Charneau P, Holloway K, Leveque M, Tricoire-Leignel H, Nicotra A, Bourdoulous S, Turowski P, Male DK, Roux F, Greenwood J, Romero IA, Couraud PO (2005). Blood-brain barrier-specific properties of a human adult brain endothelial cell line. FASEB J.

[CR38] Weller M, Rieger J, Grimmel C, Van Meir EG, De Tribolet N, Krajewski S, Reed JC, von Deimling A, Dichgans J (1998). Predicting chemoresistance in human malignant glioma cells: the role of molecular genetic analyses. Int J Cancer.

[CR39] Weller M, van den Bent M, Tonn JC, Stupp R, Preusser M, Cohen-Jonathan-Moyal E, Henriksson R, Le Rhun E, Balana C, Chinot O, Bendszus M, Reijneveld JC, Dhermain F, French P, Marosi C, Watts C, Oberg I, Pilkington G, Baumert BG, Taphoorn MJB, Hegi M, Westphal M, Reifenberger G, Soffietti R, Wick W, Associationfor Neuro-Oncology Task Force on G,  E (2017). European Association for Neuro-Oncology (EANO) guideline on the diagnosis and treatment of adult astrocytic and oligodendroglial gliomas. Lancet Oncol.

[CR40] Wick W, Gorlia T, Bendszus M, Taphoorn M, Sahm F, Harting I, Brandes AA, Taal W, Domont J, Idbaih A, Campone M, Clement PM, Stupp R, Fabbro M, Le Rhun E, Dubois F, Weller M, von Deimling A, Golfinopoulos V, Bromberg JC, Platten M, Klein M, van den Bent MJ (2017). Lomustine and bevacizumab in progressive glioblastoma. N Engl J Med.

[CR41] Yao Y, Kubota T, Takeuchi H, Sato K (2005). Prognostic significance of microvessel density determined by an anti-CD105/endoglin monoclonal antibody in astrocytic tumors: comparison with an anti-CD31 monoclonal antibody. Neuropathology : official journal of the Japanese Society of Neuropathology.

[CR42] Zhang L, Zhou F, ten Dijke P (2013). Signaling interplay between transforming growth factor-beta receptor and PI3K/AKT pathways in cancer. Trends Biochem Sci.

