# Supplementary material for: Endoglin and TGF-β signaling in glioblastoma
Source: Cell Tissue Res. 2021 Jan 20;384(3):613–24. doi: 10.1007/s00441-020-03323-5 (PMC8211614; doi:10.1007/s00441-020-03323-5)

**Supplementary Data**

**Endoglin and TGF-β signaling in glioblastoma**

Isabel Burghardt^1^, Elisa Ventura^1^, Tobias Weiss^1^, Judith Johanna Schroeder^1^, Katharina Seystahl^1^, Christian Zielasek^1^, Dorothee Gramatzki^1^ and Michael Weller^1^*

^1^Laboratory of Molecular Neuro-Oncology, Department of Neurology, University Hospital and University of Zurich, Zurich, Switzerland

*Corresponding Author: Michael Weller, MD, Laboratory of Molecular Neuro-Oncology, Department of Neurology & Brain Tumor Center, Clinical Neuroscience Center, University Hospital and University of Zurich, Frauenklinikstrasse 26, CH-8091 Zürich, Switzerland, Tel: +41442555500, Fax: +41442554507, E-mail: michael.weller@usz.ch

**Figure S1.** Individual H scores for endoglin, stratified by expression in the tumor cells or the endothelial cells are shown for two patients with available tumor tissue at time of diagnosis and time of recurrence.


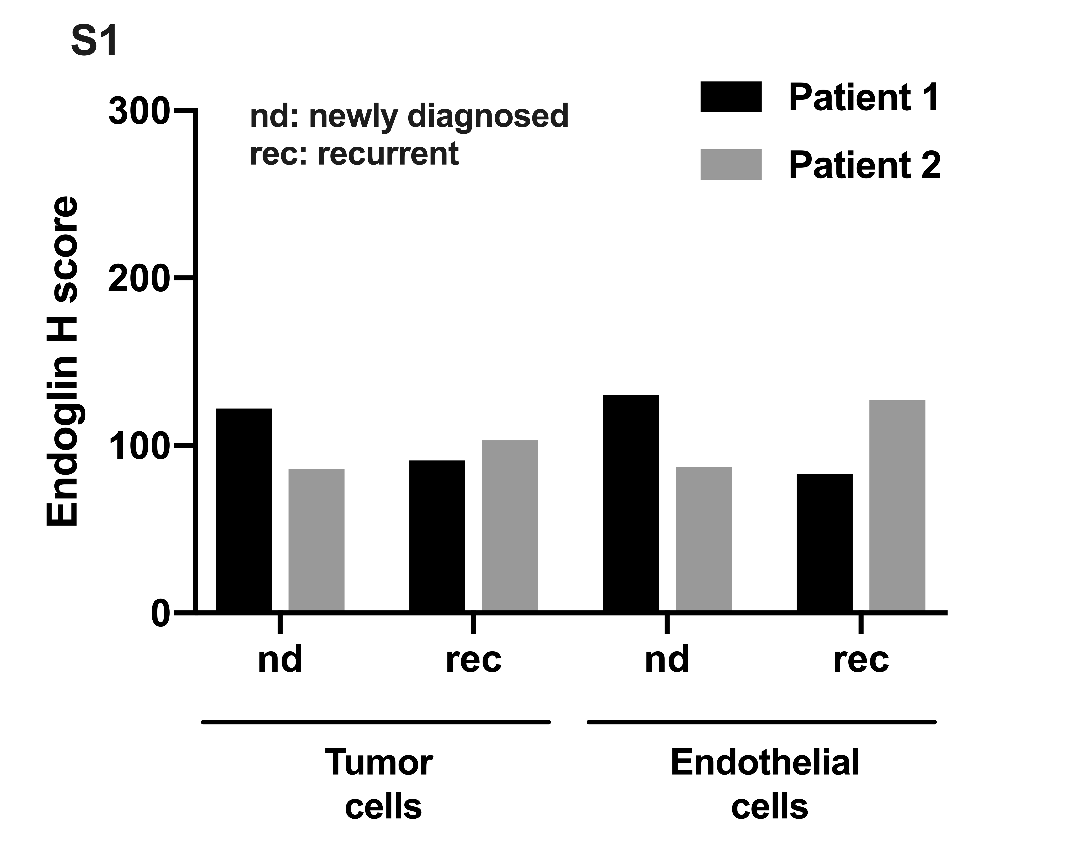


**Figure S2.** Correlation of endoglin tumor and endoglin endothelium H-score. Two-tailed Pearson test coefficients (r) and significances are indicated for newly diagnosed and recurrent tumors respectively. A linear regression analysis was performed to show a best-fit line between the two variables.


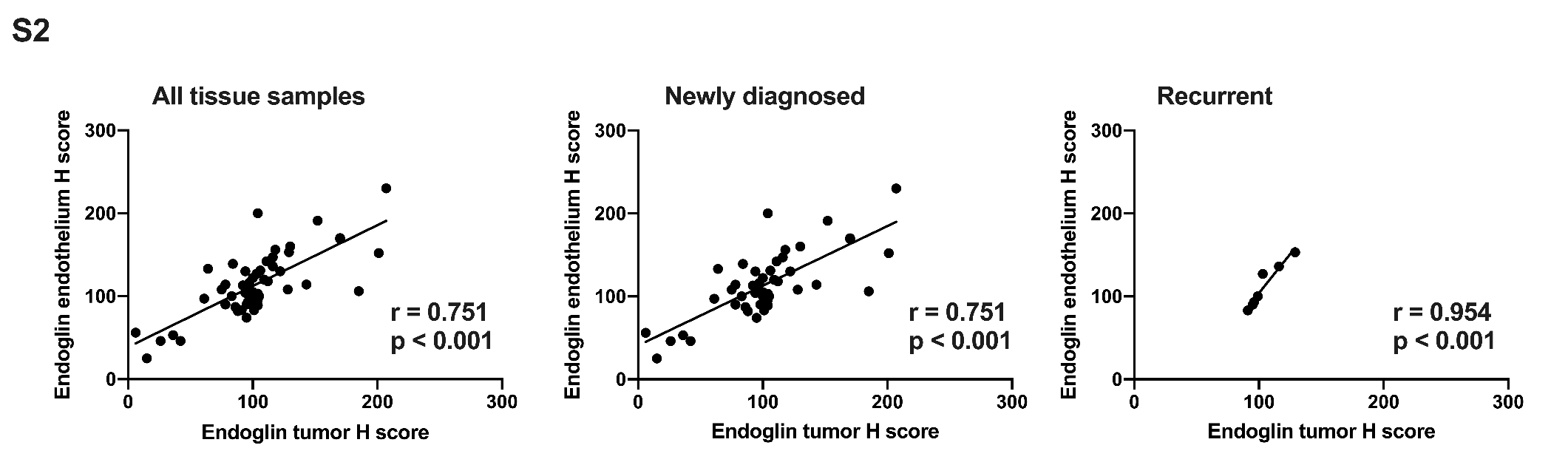


**Figure S3.** Kaplan-Meier survival curves of overall survival of patients of a glioblastoma data set of TCGA network (R2 internal identifier ps_avgpres_broadgbm540_u133a) are shown for newly diagnosed glioblastomas. Patients were divided into two groups with high (blue) or low (red) expression of the target gene with the cut-off defined by the median mRNA expression level of the target (a), alternatively, the cut-off was defined by the expression level that results in the highest association with survival (b). Statistical significances (p) were determined using the log-rank test, p< 0.05 was considered significant.


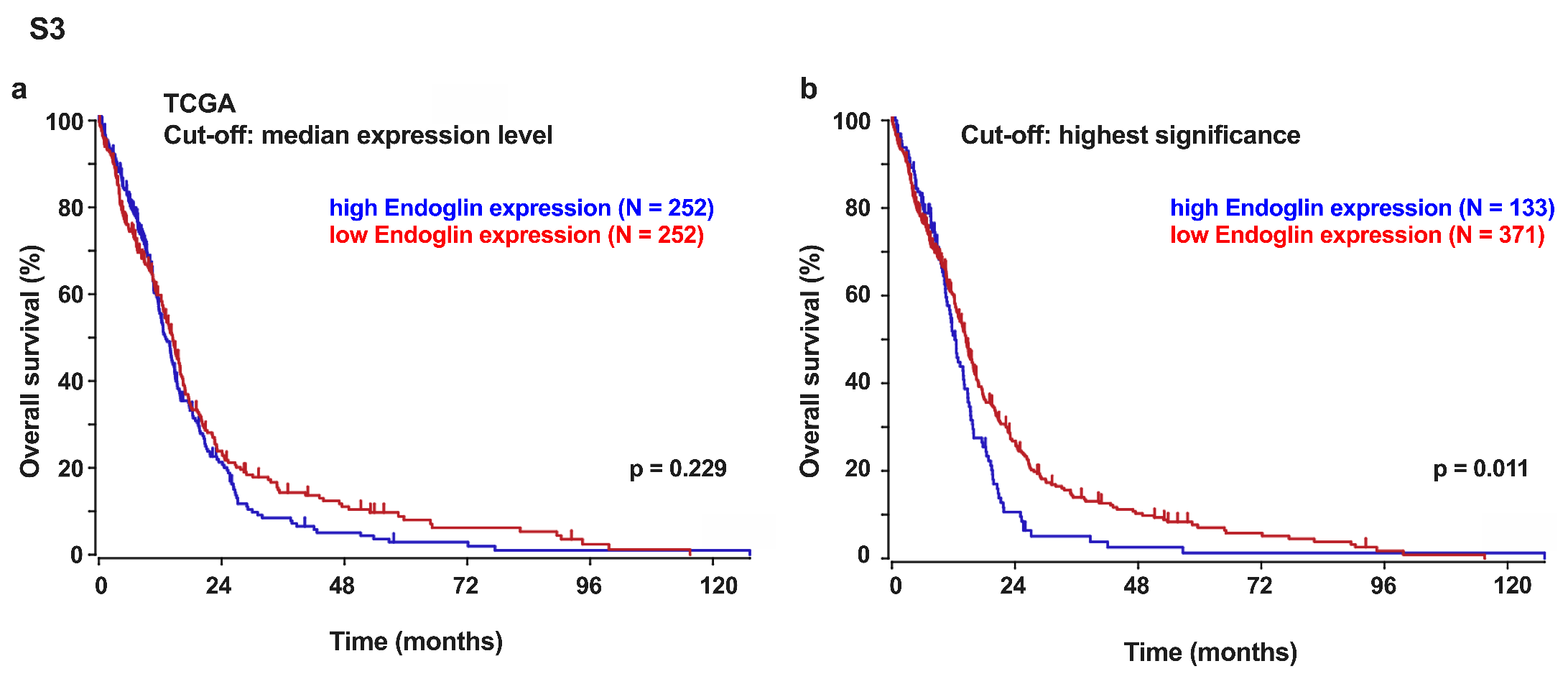


**Figure S4.** Endoglin expression correlates with expression of hypoxia-responsive genes in glioblastoma patients. Correlation analyses of mRNA expression data of *endoglin* with *CAIX, VEGFA, VEGFR1*, and *VEGFR2* are shown in glioblastoma patients from the TCGA database. Expression values are represented in a linear scale. Two-tailed Pearson test coefficients (r) and significances (p) are indicated. A linear regression analysis was performed to show a best-fit line between the two variables.

**
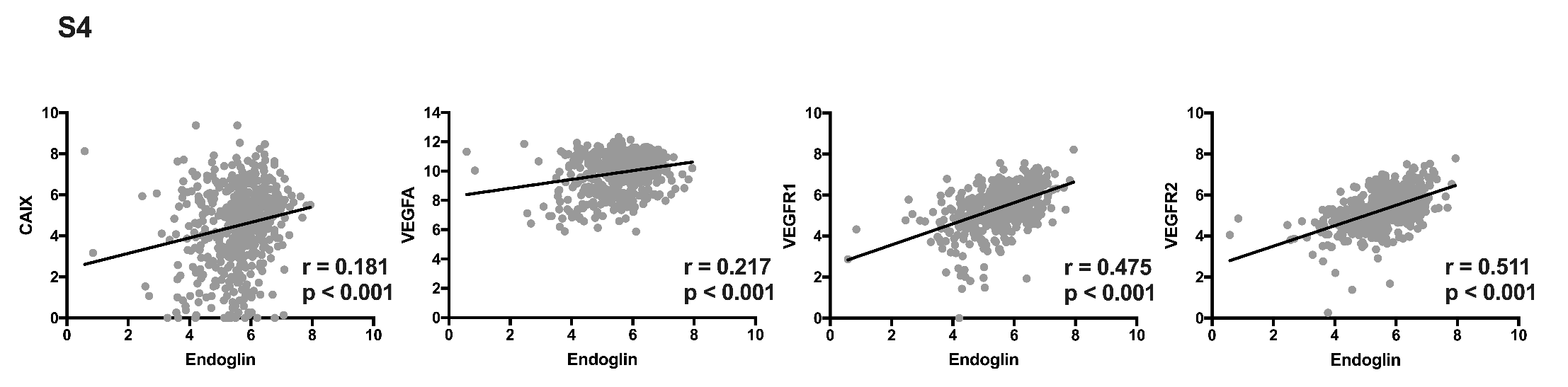
**

**Figure S5.** Endoglin expression in human glioma cell lines after culture under hypoxia. LN-18 or ZH-161 glioma cells were cultured for 24 h under hypoxia (1%) or normoxia. Subsequently, endoglin was detected by flow cytometry.

**
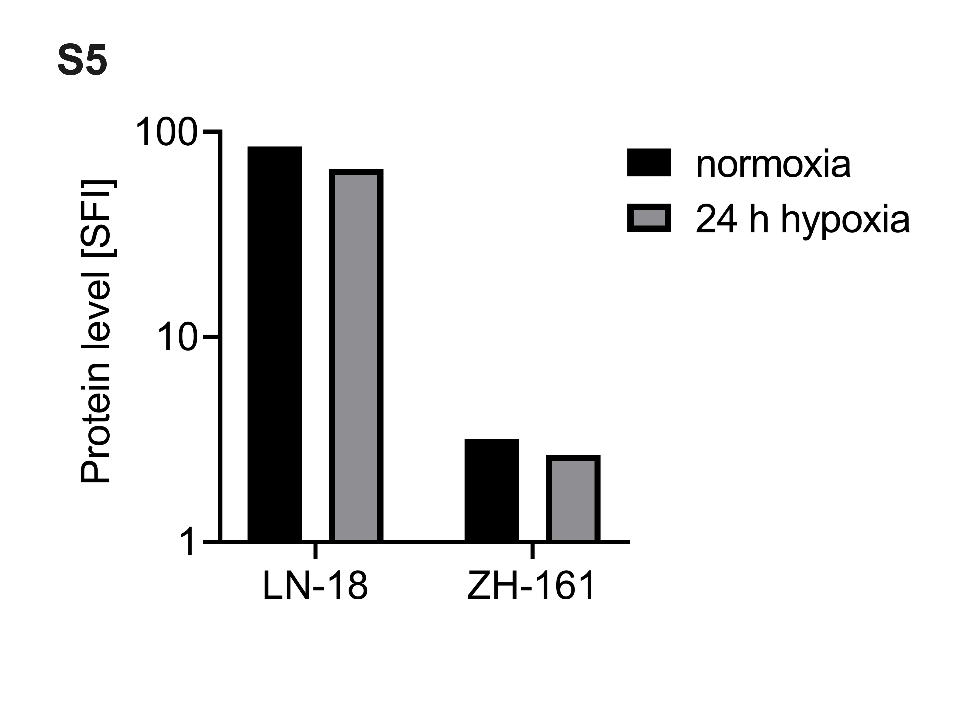
**

**Figure S6.** Distribution of CD31 and αSMA expression in cells derived from freshly dissociated human glioblastoma (n = 6). X-axis: CD31 expression value, y-axis: αSMA expression value.


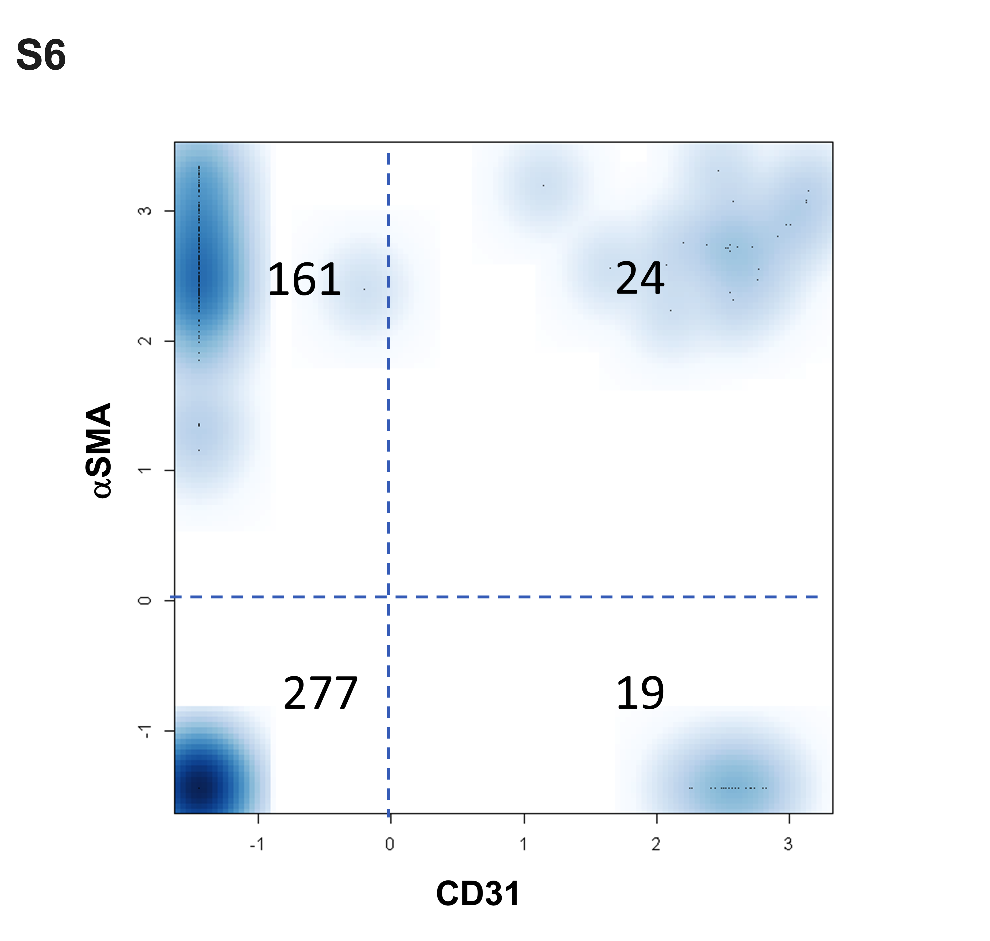

Supplement: Supplementary file 1 — Supplementary file1 (DOCX 1.1 MB) [file 441_2020_3323_MOESM1_ESM.docx]
